# An Ultrasensitive Calcium Reporter System via CRISPR-Cas9-Mediated Genome Editing in Human Pluripotent Stem Cells

**DOI:** 10.1016/j.isci.2018.10.007

**Published:** 2018-10-12

**Authors:** Yuqian Jiang, Yuxiao Zhou, Xiaoping Bao, Chuanxin Chen, Lauren N. Randolph, Jing Du, Xiaojun Lance Lian

**Affiliations:** 1 Department of Biomedical Engineering, The Pennsylvania State University, University Park, PA 16802, USA; 2 The Huck Institutes of the Life Sciences, The Pennsylvania State University, University Park, PA 16802, USA; 3 Department of Biology, The Pennsylvania State University, University Park, PA 16802, USA; 4 Department of Chemical and Biomolecular Engineering, University of California, Berkeley, CA 94720, USA; 5 College of Chemical and Biological Engineering, Zhejiang University, Hangzhou, Zhejiang 310027, China; 6 Department of Mechanical and Nuclear Engineering, The Pennsylvania State University, University Park, PA 16802, USA

**Keywords:** Cell Engineering, Optical Imaging, Specialized Functions of Cells

## Abstract

Genetically encoded calcium indicator (GCaMP) proteins have been reported for imaging cardiac cell activity based on intracellular calcium transients. To bring human pluripotent stem cell (hPSC)-derived cardiomyocytes (CMs) to the clinic, it is critical to evaluate the functionality of CMs. Here, we show that GCaMP6s-expressing hPSCs can be generated and used for CM characterization. By leveraging CRISPR-Cas9 genome editing tools, we generated a knockin cell line that constitutively expresses GCaMP6s, an ultrasensitive calcium sensor protein. We further showed that this clone maintained pluripotency and cardiac differentiation potential. These knockin hPSC-derived CMs exhibited sensitive fluorescence fluctuation with spontaneous contraction. We then compared the fluorescence signal with mechanical contraction signal. The knockin hPSC-derived CMs also showed sensitive response to isoprenaline treatment in a concentration-dependent manner. Therefore, the GCaMP6s knockin hPSC line provides a non-invasive, sensitive, and economic approach to characterize the functionality of hPSC-derived CMs.

## Highlights

• CRISPR-Cas9 was used to generate GCaMP6s expressing knockin hPSC line
• The GCaMP6 knockin cells maintain pluripotency and cardiac differentiation potential
• Knockin hPSC-derived CMs exhibit drug-responding fluorescence fluctuation
• Fluorescence fluctuation of CMs correlates with mechanical contraction signal

## Graphical Abstract

**Figure:**
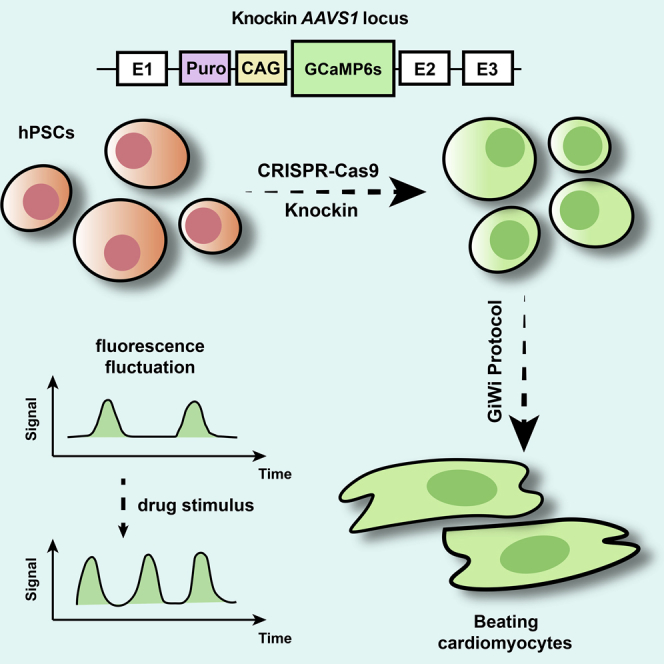

## Introduction

Human pluripotent stem cell (hPSC)-derived cardiomyocytes (CMs) hold tremendous promise for cell-based regenerative therapies, drug screening, and heart disease modeling due to the advances in stem cell differentiation research. Since hPSC-derived CMs phenotypically resemble fetal CMs in force production, adrenergic responses, and sarcomere organization (Snir et al., 2003), the functionality of hPSC-derived CMs has raised much concern for clinical applications, warranting a sensitive and accurate approach for CM characterization. Current methods for CM characterization, such as microelectrode arrays, force transducers, or calcium-sensitive dyes, cause undesirable impacts on CM contraction and are expensive and time consuming (Asakura et al., 2015, Bielawski et al., 2016, Grespan et al., 2016, Huebsch et al., 2015, Sirenko et al., 2013). Therefore, a non-invasive, cost-effective, easy-to-operate, and sensitive method for functional characterization of hPSC-CMs is needed.

Over the past decade, novel non-invasive methodologies have been developed for imaging CM activities. Among these, multi-electrode array has been used for CM research due to its scalability and reliable results (Braam et al., 2010). However, the time and cost required to produce the multi-electrode array as well as the lack of single-cell-level measurement sensitivity limit its applicability to CM research. Several force microscopy (Hazeltine et al., 2012, Pesl et al., 2016) and video microscopy (Czirok et al., 2017, Radaszkiewicz et al., 2016) methods have been developed to record and study the contractile behavior of CMs with increased accuracy and without labeling; however, they demand either specialized equipment or advanced computing software training. Similarly, acoustic and ultrasound imaging technologies (Kim and Wagner, 2016, Kunze et al., 2015) have the benefits of non-invasive and non-destructive analysis for *in vivo* or *in vitro* application but are also dependent on expensive and specialized equipment. The most commonly used non-invasive imaging modality is fluorescence microscopy, which usually requires sensitive dyes to accurately capture intracellular calcium ion fluctuation or voltage changes. The biggest challenges with this technique are the toxicity of the dyes and the resulting risk of cell malfunction caused by the chemicals, negatively affecting the reliability of results obtained using this technique. As pointed out in the most recent studies (Smith et al., 2018), chemical fluorescent Ca^2+^ indicators such as Fluo-4 acetoxymethyl (AM), Rhod-2 AM, and Fura-2 AM loaded into different cell lines suppressed their Na,K-ATPase activity, which is crucial in many physiological and pathological processes. They also altered the metabolic status, induced cell swelling, and caused a dose-dependent loss of cell viability. In contrast, the genetically encoded Ca^2+^ indicator GCaMP3 had minimal adverse effects, making it more suitable and reliable for future studies and reassessing previous observations.

GCaMP6 belongs to a family of ultrasensitive fluorescent calcium sensors, consisting of circularly permuted GFP (cpGFP), the calcium-binding protein calmodulin (CaM), and CaM-interacting M13 peptide (Chen et al., 2013). Several GCaMP variants have been developed and applied in previous studies (Hansen et al., 2017, Shinnawi et al., 2015, Zhu et al., 2014). For instance, Shinnawi et al. monitored human induced pluripotent stem cell (hiPSC)-derived CMs with genetically encoded GCaMP5G indicators (Shinnawi et al., 2015). However, the lentiviral transduction approach they used will lead to the random insertion of GCaMP into the genome, which may cause unpredictable secondary effects. To avoid this, we utilized CRISPR-Cas9 technology to ensure that GCaMP is specifically targeted to a safe harbor site for insertion. In addition, they integrated GCaMP into hiPSC-derived CMs, instead of directly into hiPSCs, limiting the further application of the generated cell line to other studies. Compared with GCamMP5s or other previous GCaMP variants, GCaMP6 sensors (GCaMP6s, 6m, and 6f; for slow, medium, and fast kinetics, respectively) exhibited higher apparent affinity for calcium and stronger saturated fluorescence, with similar baseline fluorescence. More than that, compared with the commonly used synthetic calcium dyes like OGB1-AM, GCaMP6 sensors show higher sensitivity and detect individual action potentials with high reliability at reasonable microscopic magnifications (Chen et al., 2013). For example, Dana et al. tested the application of GCaMP6s and GCaMP6f for imaging the visual cortex of transgenic mice *in vivo,* and their results demonstrated the stability and high sensitivity of the GCaMP6 system in mouse models (Dana et al., 2014). In addition, Ouzounov et al. successfully imaged the structural and functional populations of neurons in an intact mouse brain using GCaMP6s and high-resolution optical microscopy (Ouzounov et al., 2017). Despite promising results obtained with neuronal imaging, the GCaMP6 system has seen little use outside of neural applications. Due to a pervasive reliance on calcium flux to perform basic cell functions, it stands to reason that the study of other cell groups could be enhanced by the use of GCaMP6. Notably, calcium is a critical regulator of CM contractile function (Corada et al., 2013, Louch et al., 2015) making cardiovascular research an ideal field for GCaMP application. In fact, Mathur et al. recently used GCaMP6 reporter CMs for drug screening (Mathur et al., 2015). Nevertheless, the application of GCaMP6 to quantify calcium-mediated CM function remains unexplored and promises a visualized way to analyze the functional properties of hPSC-derived CMs.

Here, we report the generation of a CRISPR-Cas9-mediated GCaMP6s knockin stem cell line. These GCaMP6s knockin hPSCs can be differentiated to CMs that can be directly characterized by GFP intensity. Since no modifications are introduced to the cell surface, diastolic or systolic disturbance caused by a detective device can be avoided. The GFP intensity changes correlate with mechanical strain detected via video microscope analysis and show appropriate responses to adrenergic stimulation with isoprenaline in a concentration-dependent manner. These results highlight the ultrasensitivity and high applicability of the GCaMP6s system and establish an engineered hPSC tool for the simplified study of calcium-dependent cell functionality.

## Results

### CRISPR-Cas9-Mediated Generation of GCaMP6s Knockin Cell Line

We previously utilized gene overexpression or knockdown approaches in hPSCs to study gene function during stem cell differentiation (Lian et al., 2012). We have also used lentiviral or PiggyBac strategies to integrate our designed DNA constructs into the genome of hPSCs to study the important role of the Wnt signaling pathway for stem cell differentiation (Lian et al., 2012, Randolph et al., 2017). However, the randomly inserted DNA constructs are susceptible to silencing during stem cell differentiation. To achieve efficient and precise gene editing, we employed CRISPR-Cas9 technology. We first cloned our previously described, all-in-one DNA elements (Randolph et al., 2017) into an *AAVS1* safe harbor knockin plasmid (Figure S1A). To obtain stable gene expression, it is essential to precisely insert foreign DNA constructs to a safe harbor locus, such as AAVS1, which will not be silenced during stem cell proliferation and differentiation (Qian et al., 2014). Upon doxycycline treatment and before fluorescence-activated cell sorting (FACS) sorting, 27% of the cells showed GFP expression (Figure S1B), indicating that the CRISPR-Cas9 system can be employed to generate knockin expression cassettes at the *AAVS1* safe harbor locus of the human genome efficiently. Successfully modified cells (GFP+) were isolated via FACS. Consistent with our previous report (Randolph et al., 2017), sorted hPSCs did not show any GFP expression without doxycycline treatment, and all cells appeared to express GFP upon doxycycline exposure post-sorting (Figure S1C).

**Figure 1:**
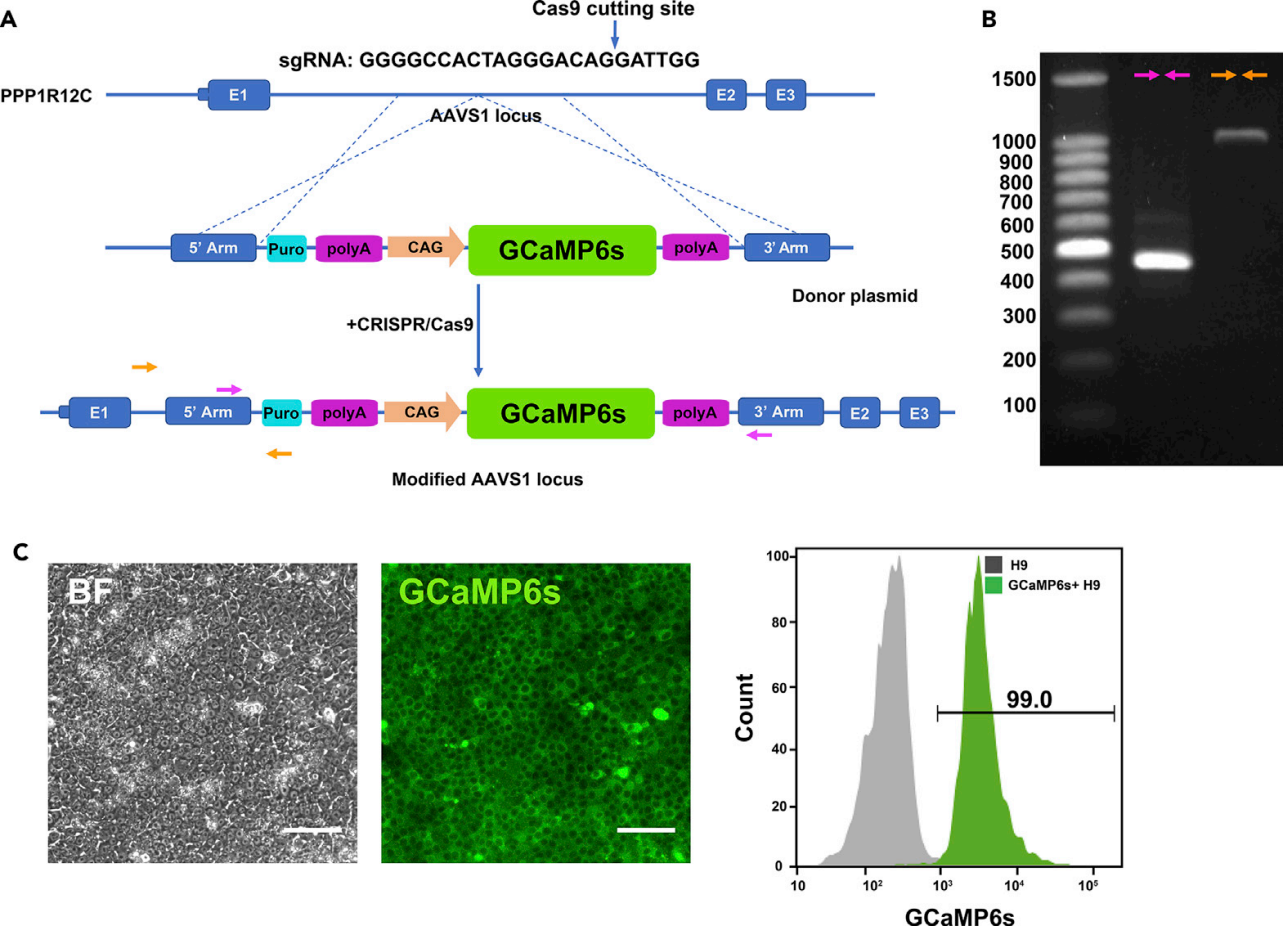
Generation of GCaMP6s Knockin hPSCs (A) Schematic of GCaMP6s knockin with CRISPR-Cas9. Under the guidance of designed single guide RNA (sgRNA), Cas9 cleaved the target site in the genome of H9 cells and GCaMP6s sequence was recombined into the *AAVS1* locus between exon 1 and exon 2 of the *PPP1R12C* gene in chromosome 19. Primer 1 (pink) and primer 2 (orange) were designed to confirm heterozygous knockin. (B) Gel electrophoresis of PCR products with two pairs of primers. Bands of 479 bp with primer 1 and 1 kb with primer 2 indicated heterozygous knockin. (C) Representative phase-contrast and GFP epifluorescence images (left) and flow cytometry analysis (right) of GCaMP6s knockin H9 cells. Regular H9 was used as control. Scale bar, 100 μm. See also Figure S1.

Fluorescent calcium sensors are widely used to image neural activity (Chen et al., 2013, Li et al., 2003, Ohkura et al., 2012). Similarly, to visualize cardiac calcium activity, we decided to engineer a hPSC line (H9) to constitutively express the ultrasensitive fluorescent protein GCaMP6s (Chen et al., 2013) at the *AAVS1* safe harbor locus via Cas9-mediated homologous recombination (Figure 1A). After puromycin selection, we picked GCaMP6s-positive single-cell-derived hPSC clones and performed genotyping to confirm the success of targeted knockin (Figure 1B). We designed two sets of primers to test whether we had a successful knockin clone with the GCaMP6s expression cassette at the *AAVS1* locus. We also performed live cell imaging and flow cytometry analysis of our engineered cells (Figure 1C).

To confirm the knockin (Figure 1A), the forward and reverse oligos of primer set 1 were located in the 5′ and 3′ arms of the donor plasmid, respectively. A wild-type allele would generate a 479-bp band, whereas successful knockin of GCaMP6s expression cassette would generate a 7-kb PCR product, which is too long to be synthesized by DNA polymerase due to the limited elongation time specified in our PCR conditions. For primer set 2, the forward oligo was designed upstream of the 5′ arm in the genome and the reverse oligo was located within the donor construct (between the 5′ arm and the puromycin sequence). With this design, a successful GCaMP6s knockin clone would show a 1-kb band, otherwise there would be no band observed. PCR results shown in Figure 1B demonstrated the existence of a wild-type allele as well as a GCaMP6s knockin allele, indicating the generation of a heterozygous knockin cell line.

**Figure 2:**
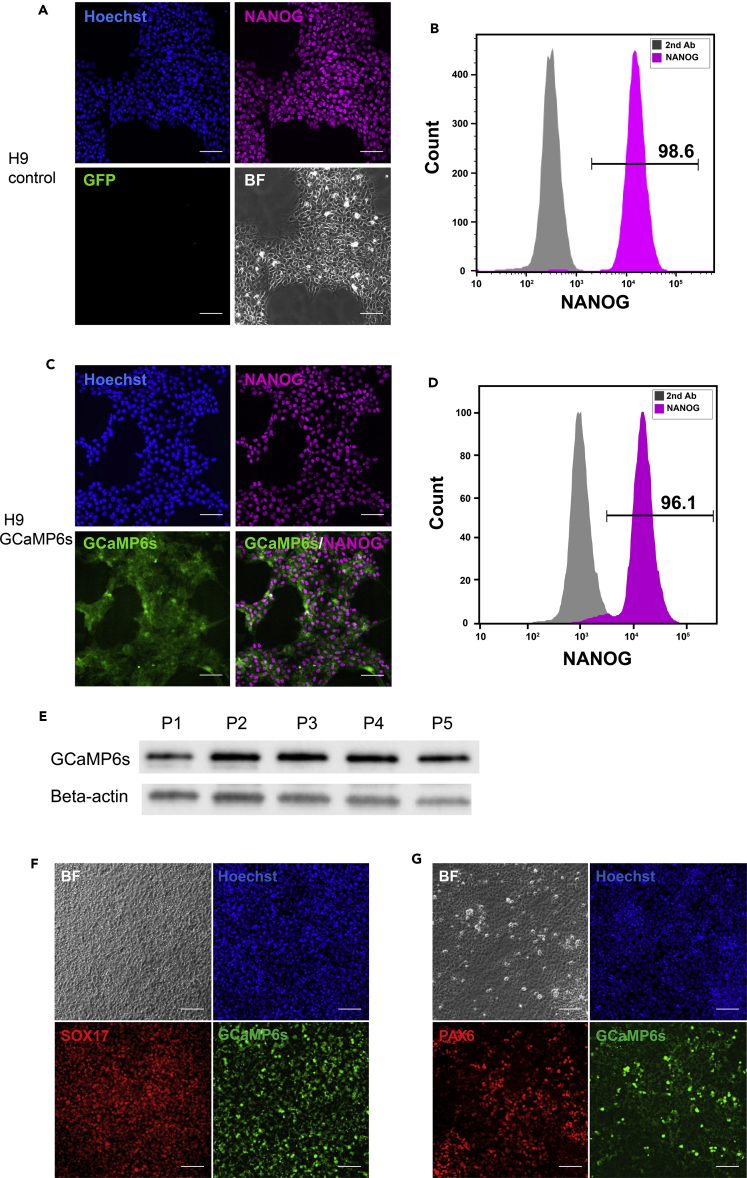
The GCaMP6s Knockin Cell Line Remains Pluripotent (A and B) Regular H9 cells were analyzed via immunofluorescence (A) and flow cytometry (B) for pluripotency marker NANOG. Scale bar, 100 μm. (C and D) GCaMP6s knockin H9 cells were analyzed via immunofluorescence (C) and flow cytometry (D) for pluripotency marker NANOG. Scale bar, 100 μm. Over 95% of the knockin cells expressed pluripotency markers. Control: cells stained with second antibody only. (E) Expression of GCaMP6s was analyzed via western blot in GCaMP6s knockin H9 cells undergoing 5 passages. β-Actin was used as loading control. (F) GCaMP6s knockin H9 cells were differentiated into endoderm cells by 100 ng/mL activin A treatment for 4 days. Expression of Sox17 was analyzed via immunofluorescence. Scale bar, 100 μm. (G) GCaMP6s knockin H9 cells were differentiated into ectodermal cells by LaSR basal medium treatment for 6 days. Expression of PAX6 was analyzed via immunofluorescence. Scale bar, 100 μm. See also Figure S2.

Since calcium signaling is ubiquitously involved in intracellular signaling that controls numerous cellular processes such as cell differentiation, proliferation, and apoptosis (Resende et al., 2010, Tonelli et al., 2012), green fluorescence from constitutive expression of GCaMP6s was observed in the generated knockin cell line via fluorescent microscopy, and also by flow cytometry (Figure 1C), further confirming successful knockin. To verify the pluripotency of the generated cell line, we chose markers of pluripotency NANOG and SSEA4 for immunostaining and flow analysis. Our results showed that the GCaMP6s knockin H9 cells expressed pluripotent markers NANOG (Figures 2A–2D) and SSEA4 (Figures S2A–S2D), similar to regular H9 cells. To characterize the cell viability of our knockin cells and non-engineered cells, we performed a trypan blue assay. Our results showed that cell viability reached 95% for both non-engineered cells and our GCaMP6s knockin cells. To quantify whether GCaMP6s expression level changes over time, we collected GCaMP6s knockin cells over 5 passages and we did not observe strong up-regulation of GCaMP6s expression over time (Figure 2E). Furthermore, GCaMP6s knockin cells were successfully differentiated into SOX17+ endoderm cells (Figure 2F) and PAX6+ ectoderm cells (Figure 2G).

### Analysis of GCaMP6s Knockin hPSC-Derived CMs

**Figure 3:**
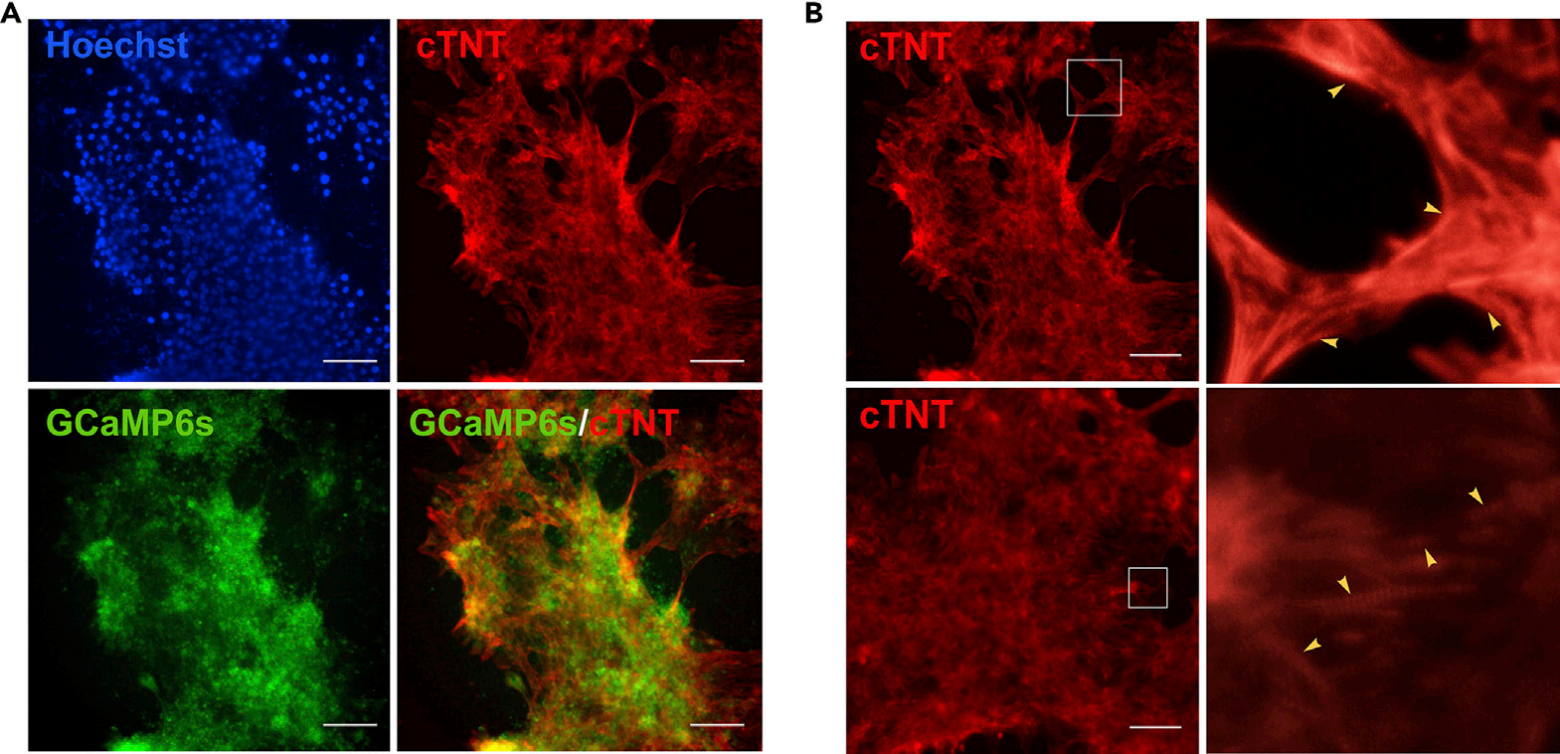
The GCaMP6s Knockin hPSCs Generated Cardiomyocytes (A) Cardiomyocytes were generated from GCaMP6s knockin H9 cells using the GiWi protocol with CHIR99021 and Wnt-C59 treatment. Immunofluorescence analysis with cardiac markers cTNT and GCaMP6s expression. Scale bar, 100 μm. (B) Immunostaining for cTNT shows sarcomeric striations (yellow arrowheads). See also Figure S3.

To investigate the efficacy of using the GCaMP6s reporter system for CM functional characterization, directed differentiation of GCaMP6s knockin hPSCs to CMs was performed with our previously reported GiWi protocol (Lian et al., 2012). Immunostaining against cardiac-specific marker cardiac troponin T (cTNT) on day 30 verified the cardiac differentiation potential of the knockin cell line (Figure 3A). Overlap of GCaMP6s fluorescence with cTNT (Figure 3A) or myosin heavy chain (Figures S3A and S3B) indicated that CMs derived from the knockin hPSC line retained GCaMP6s activity. Immunostaining for cTNT shows sarcomeric striations (Figure 3B) present in CMs. Furthermore, quantification of CM differentiation efficiency demonstrated that the GCaMP6s knockin cells did not show less differentiation efficiency than non-engineered cells (Figure S3C).

**Figure 4:**
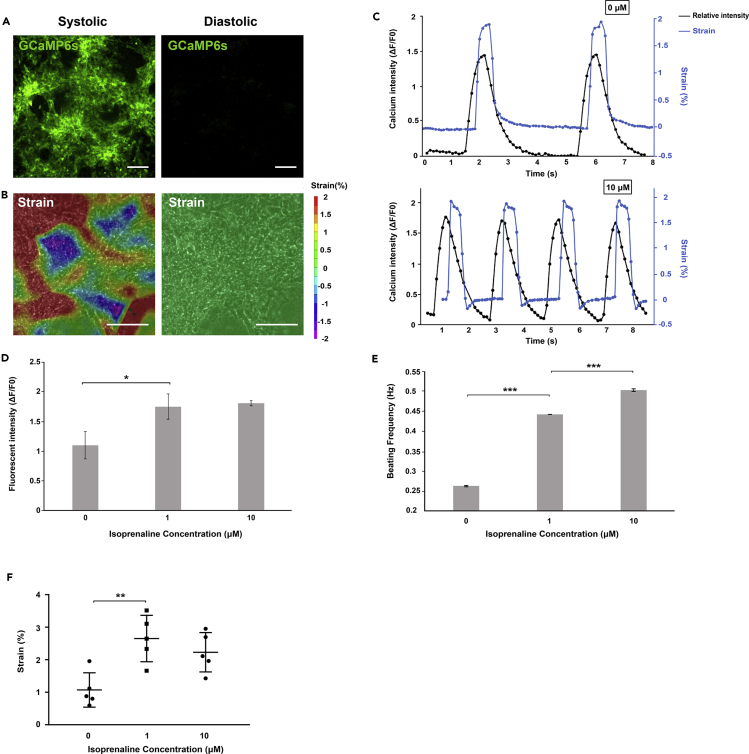
The GCaMP6s Knockin hPSC-Derived Cardiomyocytes Responded to Isoprenaline Treatment (A and B) Difference in fluorescence intensity (A) and contractile strain (B) between systolic (left) and diastolic (right) status. Scale bar, 100 μm. (C) Fluctuation of fluorescence intensity and strain of CMs treated with 0 and 10 μM isoprenaline. (D–F) Fluorescence intensity (D), beating frequency (E), and strain (F) of CMs treated with varied isoprenaline concentrations from 0 to 10 μM (n = 5 for each group; * 0.05 < p value < 0.1, **0.01 < p value < 0.05, ***p value < 0.01, mean ± SD). See also Figure S4.

Next, we quantified GCaMP6s fluorescence intensity during CM contraction cycles. CMs presented distinct fluorescence intensities in systolic and diastolic states, respectively (Figure 4A; Videos S1, S2, S3, S4, S5, and S6), enabling the correlation of the spontaneous contraction via cyclic fluorescence intensity fluctuations.

Video S1. Bright-Field Video of Cardiomyocytes Derived from GCaMP6s Knockin Cells without Drug Treatment, Related to Figure 4

Video S2. Fluorescence Video of Cardiomyocytes Derived from GCaMP6s Knockin Cells without Drug Treatment, Related to Figure 4

Video S3. Bright-Field Video of Cardiomyocytes Derived from GCaMP6s Knockin Cells with 1 μM Isoprenaline Treatment, Related to Figure 4

Video S4. Fluorescence Video of Cardiomyocytes Derived from GCaMP6s Knockin Cells with 1 μM Isoprenaline Treatment, Related to Figure 4

Video S5. Bright-Field Video of Cardiomyocytes Derived from GCaMP6s Knockin Cells with 10 μM Isoprenaline Treatment, Related to Figure 4

Video S6. Fluorescence Video of Cardiomyocytes Derived from GCaMP6s Knockin Cells with 10 μM Isoprenaline Treatment, Related to Figure 4

To verify the fidelity of fluorescence-based cell characterization, we also analyzed the selected locations in captured videos to determine the mechanical behaviors of the CMs (Figure 4B). The results showed that, in each contraction cycle, there were several regions in contraction, as well as several areas in dilation, in the CM clusters. We assumed there were cells with different functions in the cluster, some of which were more able to contract. When these cells contracted, the other cells with lower contractile ability were pulled and resulted in dilation in those regions. The beating motion (strain over time) of CMs was also obtained (Figure 4C) from digital image correlation. We found that the contractile motions presented similar frequency to the frequency of fluorescence intensity fluctuation in the same CMs.

### GCaMP6s Knockin hPSC-Derived CMs Respond to Adrenergic Stimulus

To test the functional sensitivity of CMs derived from the generated cell line, we tested their response to a commonly used pharmacological agent. Isoprenaline is a non-selective β-adrenoreceptor agonist and is commonly used for the treatment of conditions in which increased heart function is required, such as bradycardia, heart block, and congestive heart failure. The effects of isoprenaline on the cells were observed as increased contractile activity of the CMs, and the beating frequency was measured by taking videos of the GFP expression of beating CMs with fluorescence microscopy. The fluctuations of fluorescent intensity in the videos were analyzed in ImageJ.

Upon treatment with isoprenaline, the fluorescent intensity fluctuation and contractile motion for CMs changed in terms of amplitude and frequency (Figures 4C and S4). Calcium signals appeared 0.33 and 0.44 s ahead of mechanical signals, for 0 and 10 μM isoprenaline treatment, respectively, illustrating the causal relationship between intracellular calcium activation and the beginning of systole. We observed that the amplitude for normalized fluorescence intensity (Figure 4D) significantly increased from 1.104 before drug administration to 1.752 at 1 μM isoprenaline addition. However, it did not significantly change from 1 to 10 μM isoprenaline (with 1.809 normalized intensity). The amplitude of contractile motions (strain) (Figure 4F) also significantly increased from 1.07% before drug administration to 2.65% at 1 μM isoprenaline addition. It did not significantly change from 1 to 10 μM isoprenaline (2.23%). In addition, mechanical analysis showed a statistically significant increase in contractile strain with isoprenaline administration, consistent with previously reported results regarding cardiac cell strain change with adrenergic agonists (Hansen et al., 2017).

Furthermore, frequency analysis (Figure 4E) indicated that isoprenaline treatment caused accelerated contraction with a statistically significant increase in beating frequency from 0.263 Hz before drug administration to 0.443 Hz at 1 μM. The contraction further accelerated significantly from 0.443 Hz at 1 μM to 0.504 Hz at 10 μM isoprenaline addition.

Following drug treatment, immediate isoprenaline removal, and overnight incubation, cells presented an abnormal contractile phenotype and severe detachment, which usually occurs in long-time cultured CMs. One possible reason for this reaction might be the limited capacity of hPSC-derived CMs to degrade isoprenaline due to their immature phenotype (Hansen et al., 2017).

## Discussion

With the rapid advances in stem cell technology, hPSC-derived CMs hold great promise in a wide variety of applications, including myocardial regeneration therapies, disease modeling, and drug screening. To reach the potential of these applications, cost-effective and reliable approaches for CM characterization are required. For this purpose, we generated a calcium reporter stem cell line by inserting GCaMP6s into the genome utilizing CRISPR/Cas9 tools, enabling us to concurrently monitor physiological properties such as contractile strain or transient calcium flux in hPSC-derived single CMs or CM clusters.

Compared with previous techniques for CM characterization, our generated reporter cell line presents tremendous advantages by overcoming challenges to existing methods. First, approaches involving calcium dyes or electrode patches inevitably lead to extrinsic chemical or physical perturbations, which can adversely affect the results of cellular characterization. In contrast, genomic insertion of the sequence of a calcium reporter protein into stem cells allows consistent functional evaluation from the starting line over all developmental stages from pluripotent to differentiated somatic cells. Furthermore, our genetically engineered cell line theoretically and experimentally avoids foreign disturbance or cell sacrifice, unlike in terminal analysis techniques, providing a non-invasive and simple method for cell function characterization based on calcium indication.

Our GCaMP6s system is ultrasensitive on both protein and cellular scales. We chose to knockin GCaMP6s due to its higher sensitivity over commonly used synthetic calcium dyes, such as OGB1-AM, and its ability to detect individual action potentials with increased reliability at reasonable microscope magnifications (Chen et al., 2013). At the cellular level, the CMs derived from the GCaMP6s knockin hPSCs show a statistically significant change in beating frequency with isoprenaline treatment as low as 1 μM, exhibiting the high functional sensitivity of this cell line. For analysis, fluorescence microscopy further increases the resolution up to single-cell observation.

Operational convenience is another advantage of our reporter system. For construction of the knockin cell line, CRISPR/Cas9 technology has made it more efficient and reliable to perform targeted genome editing. For stem cell differentiation, pivotal regulating pathways have been gradually uncovered in developmental biology, generating more straightforward differentiation protocols and making *in vitro* stem cell differentiation increasingly precise. For calcium indication, visualization of calcium signaling transforms chemical and electrical signals inside cells to detectable fluorescent signals, enabling real-time data acquisition and analysis. Last but not least, the differentiation potential of hPSCs makes it possible for this calcium-indicating reporter system to be applied in the characterization of other cell lineages, such as neurons or muscle cells, which can further contribute to disease modeling and drug screening in the extended range.

In summary, the GCaMP6s knockin stem cell line provides an ultrasensitive, non-invasive, reliable, convenient, and economic calcium indicator system, which can be utilized for, but is not limited to, functional characterization of hPSC-derived CMs.

### Limitations of the Study

Despite the convenience and sensitivity of our designed knockin stem cell line, this system can be further modified in several aspects. First, constitutive expression of GCaMP6s protein in the current design can be replaced by inducible expression with the Tet-On system. In this case, GCaMP6s will be expressed only when cells accomplish the desired differentiation and are ready for characterization, which would avoid potential cell toxicity caused by protein accumulation during the differentiation process. In addition, similar knockin approach with genetically encoded membrane voltage indicator can also be investigated that would minimize the interference of intracellular calcium ion equilibrium. Last, here we only applied our knockin hPSC line for CM characterization. Performance of this reporter system for other cell lineages can be further tested.

## Methods

All methods can be found in the accompanying Transparent Methods supplemental file.

## Supplemental Information

Document S1. Transparent Methods and Figures S1–S4
