# Supplementary material for: An Ultrasensitive Calcium Reporter System via CRISPR-Cas9-Mediated Genome Editing in Human Pluripotent Stem Cells
Source: iScience. 2018 Oct 12;9:27–35. doi: 10.1016/j.isci.2018.10.007 (PMC6203247; doi:10.1016/j.isci.2018.10.007)
Supplement: Document S1. Transparent Methods and Figures S1–S4 [file mmc1.pdf]

ISCI, Volume *9*

Supplemental Information

An Ultrasensitive Calcium Reporter System via CRISPR-Cas9-Mediated Genome Editing in Human Pluripotent Stem Cells

Yuqian Jiang, Yuxiao Zhou, Xiaoping Bao, Chuanxin Chen, Lauren N. Randolph, Jing Du, and Xiaojun Lance Lian

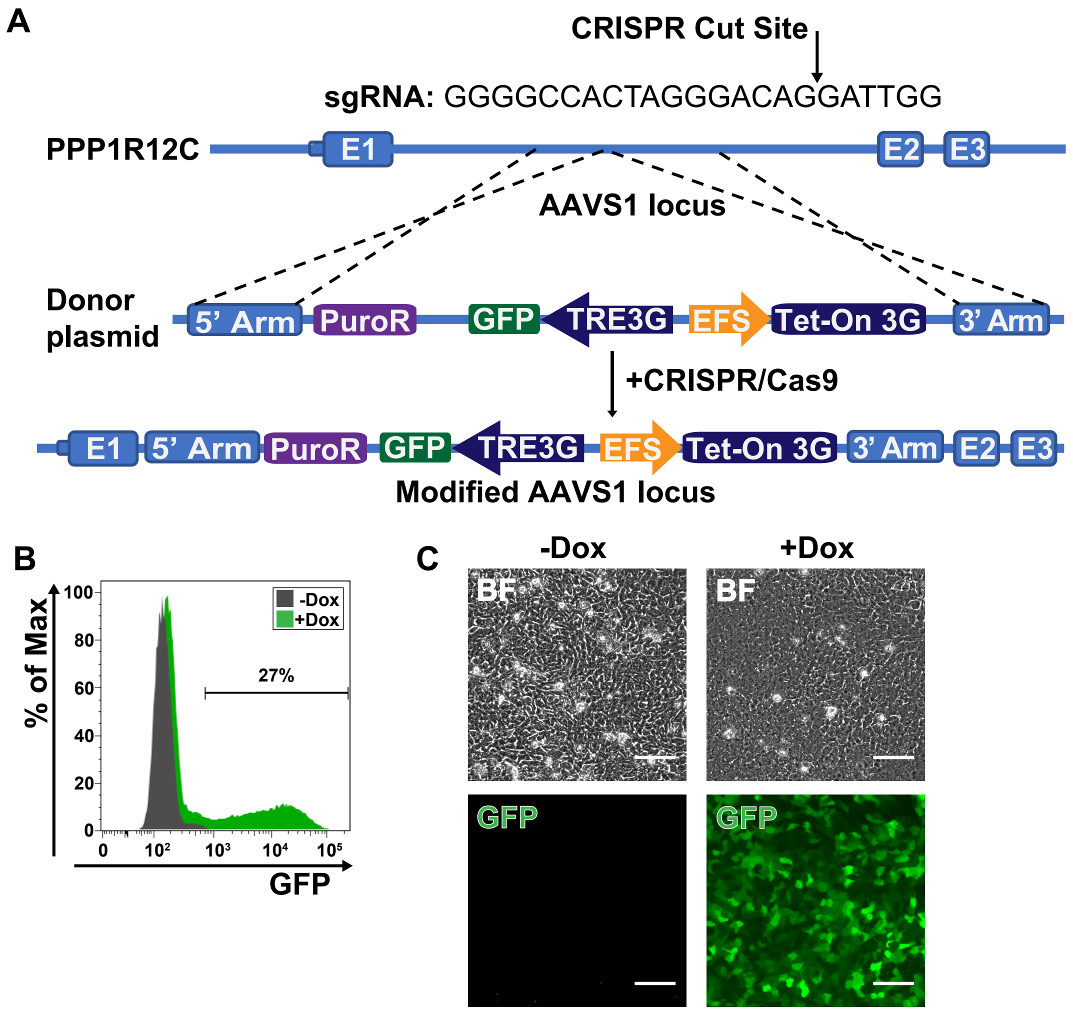

Figure S1. Generation of XLone-GFP knockin hPSCs, Related to Figure 1. (A) Schematic of the knockin design to insert the donor plasmid containing XLone-GFP into the AAVS1 locus. (B) Flow cytometry analysis showing 27% of the population becomes GFP positive upon exposure to 2 μg/mL doxycycline for 24 hours. (C) Images showing GFP expression of sorted cells not treated and treated with doxycycline at 2 μg/mL for 24 hours. Scale bars are 100 μm.

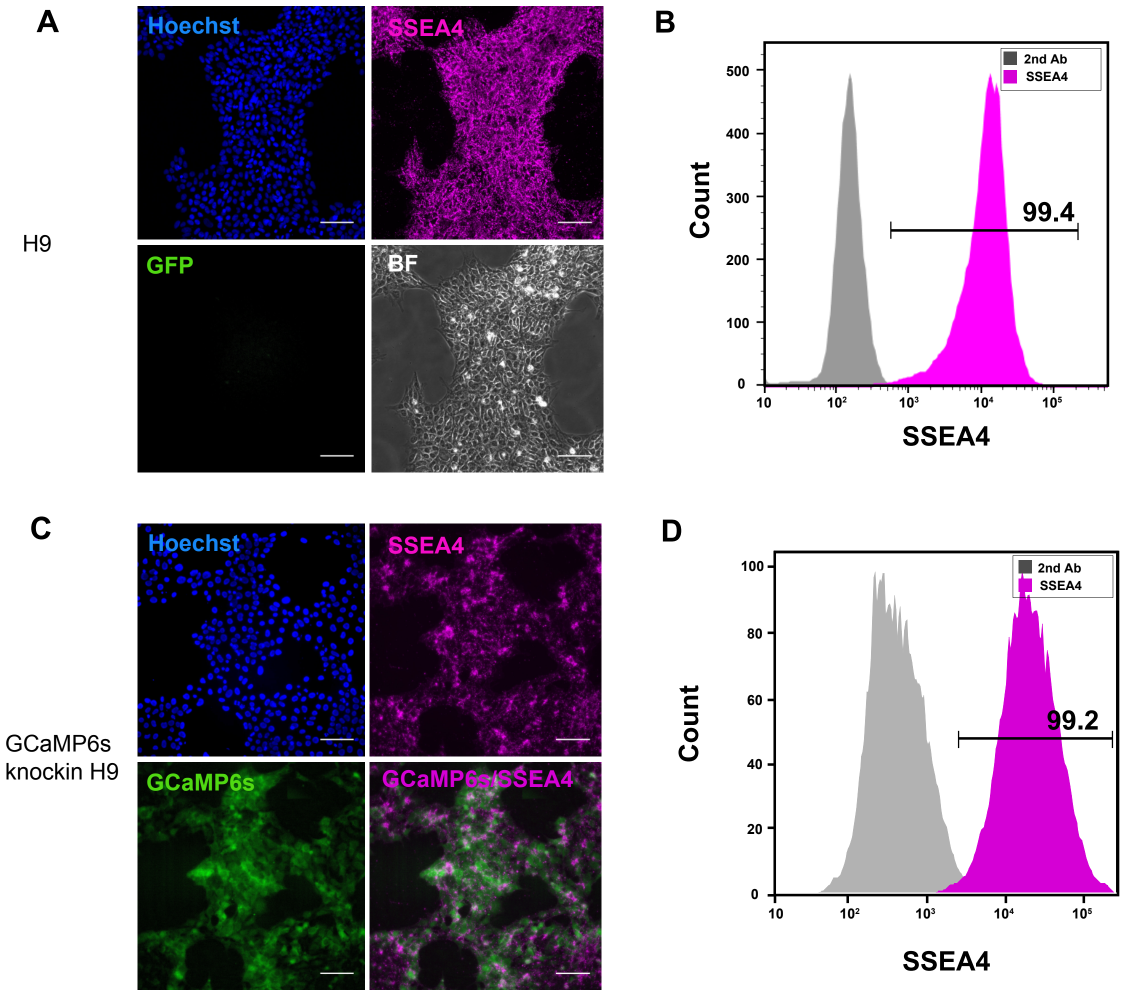

Figure S2. The GCaMP6s knockin cell line remains pluripotent, Related to Figure 2. (A and B) Regular H9 cells were analyzed via immunofluorescence (A) and flow cytometry (B) for pluripotency marker SSEA4. Scale bar: 100 μm. (C and D) The GCaMP6s knockin H9 cells were analyzed via immunofluorescence (C) and flow cytometry (D) for pluripotency marker SSEA4. Scale bar: 100 μm. Control: cells stained with second antibody only. SSEA4: Stage-specific embryonic antigen 4.

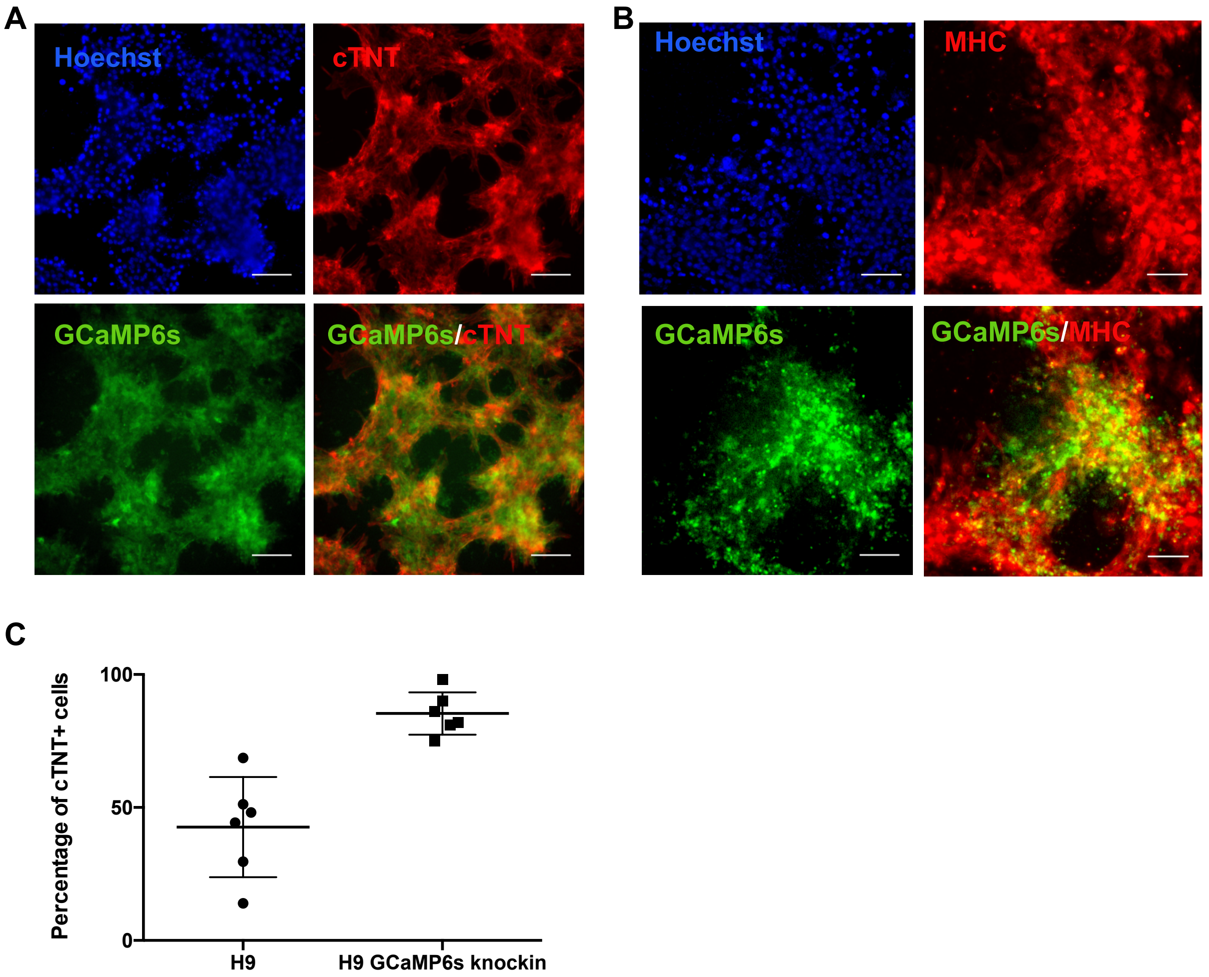

Figure S3. Characterization of cardiomyocytes derived from the GCaMP6s knockin H9 cells, Related to Figure 3. (A and B) Cardiomyocytes were generated from GCaMP6s knockin H9 cells using the GiWi protocol with CHIR99021 and Wnt-C59 treatment. Immunofluorescence analysis with cardiac markers cTNT (A) and MHC (B) expression. Scale bar: 100 μm. cTNT: cardiac troponin T. MHC: myosin heavy chain. (C) Quantitative analysis of the percentage of cTNT+ cells generated from regular H9 cells and the GCaMP6s knockin cells.

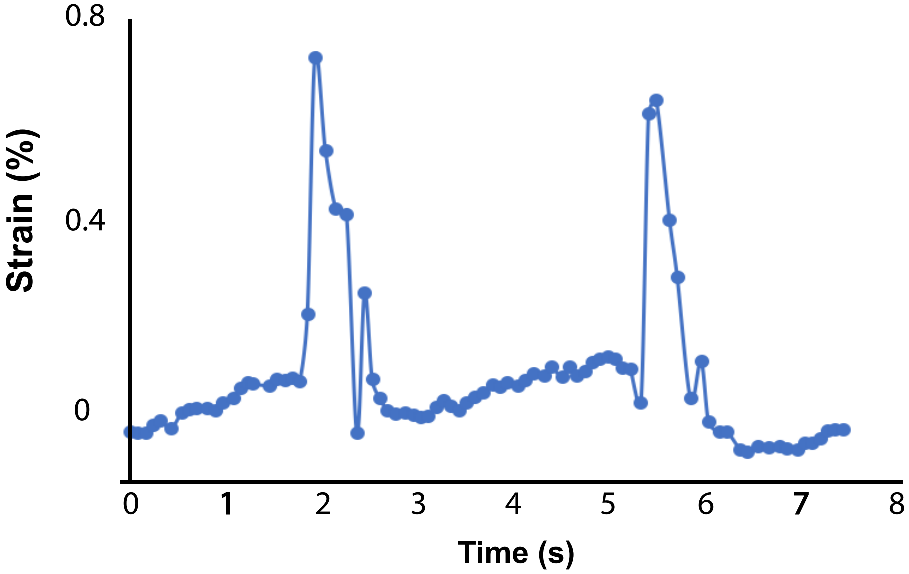

Figure S4. The GCaMP6s knockin hPSC derived cardiomyocytes responded to isoprenaline treatment, Related to Figure 4. Representative strain data of cardiomyocytes without isoprenaline treatment.

Movie S1. Bright field video of cardiomyocytes derived from GCaMP6s knockin cells without drug treatment, Related to Figure 4.

Movie S2. Fluorescent video of cardiomyocytes derived from GCaMP6s knockin cells without drug treatment, Related to Figure 4.

Movie S3. Bright field video of cardiomyocytes derived from GCaMP6s knockin cells with 1 μM isoprenaline treatment, Related to Figure 4.

Movie S4. Fluorescent video of cardiomyocytes derived from GCaMP6s knockin cells with 1 μM isoprenaline treatment, Related to Figure 4.

Movie S5. Bright field video of cardiomyocytes derived from GCaMP6s knockin cells with 10 μM isoprenaline treatment, Related to Figure 4.

Movie S6. Fluorescent video of cardiomyocytes derived from GCaMP6s knockin cells with 10 μM isoprenaline treatment, Related to Figure 4.

Antibody

	Primary antibody		Secondary antibody	
Oct3/4	Mouse IgG2b, Santa Cruz	1:100	Anti-mouse IgG2b, Alexa Fluo 633	1:1000
Nanog	Rabbit IgG, Thermo Fisher Scientific	1:500	Anti-rabbit IgG, Alexa Fluo 647	1:1000
SSEA4	Mouse IgG3, DSHB	1:20	Anti-mouse IgG, Alexa Fluo 647	1:1000
cTNT	Mouse IgG1, Thermo Fisher Scientific	1:200	Anti-mouse IgG1, Alexa Fluo 647	1:1000
MF20 (MHC)	Mouse IgG2b, DSHB	1:30	Anti-mouse IgG2b, Alexa Fluo 633	1:1000
SOX17	Human Sox17 APC-conjugated antibody, R&D systems			1:50
PAX6	Mouse IgG1, DSHB	1:50	Anti-mouse IgG1, Alexa Fluo 647	1:1000

Primer information (Related to Figure 1**)**

Primer_1_F: TGCTTTCTTTGCCTGGACAC

Primer_1_R: GGTTCTGGCAAGGAGAGAGA

Primer_2_F: CCATAGCTCAGGTCTGGTCTAT

Primer_2_R: AGGAAGAGAAGAGGTCAGAAGC

Transparent Methods

Maintenance of hPSCs

Human embryonic stem cells (H9 line) maintained on Matrigel (Corning) in mTeSR1 (STEMCELL Technologies) were dissociated into single cells with 0.5 mM EDTA at 37°C for 10 min and then were seeded onto a new Matrigel-coated cell culture dish at 10,000-20,000 cell/cm^2 in mTeSR1 supplemented with 5 μM ROCK inhibitor (Y-27632) for 24 h. Cells then were cultured in mTeSR1, which was changed daily. Human embryonic stem cell research was approved by the Pennsylvania State University Embryonic Stem Cell Oversight Committee.

Generation of AAVS1 GCaMP6s knockin hPSC line

hPSCs were pre-treated with 10 μM Y27632 for 4 hours or overnight prior to electroporation. Cells were digested by StemPro Accutase (Thermo Fisher) at 37°C for 8 min and 2.5-3 million single cells were electroporated with 4 μg pCas9_GFP (Addgene plasmid # 44719), 2.5 μg gRNA T2 (Addgene plasmid # 41818) and 6 μg AAVS1 CAG Flag-MAR-P2A GCaMP6s donor plasmid in 200 μL cold PBS -/- using the Gene Pulser Xcell System (Bio-Rad) at 320 V, 200 μF and 1000 Ω (Time constant is ~15 ms) in a 0.4 cm cuvette. To generate the AAVS1-pur-CAG Flag-MAR P2A GCaMP6s donor plasmid, we directly synthesized Flag-MAR (Integrated DNA Technologies), amplified GCaMP6s by PCR from pGP-CMV-GCaMP6s (Addgene # 40753), and inserted both fragments into the AAVS1-puro-CAG-hrGFP plasmid (Addgene plasmid #52344) replacing GFP. The resulting cells were subsequently plated onto a Matrigel-coated 6-cm dish in 5 mL mTeSR1 with 10 μM Y27632. 24 hours later, and every day afterwards, the medium was changed with fresh mTeSR1. Three days after electroporation, 1 μg/ml puromycin was added into the mTeSR1 for selection for about two weeks. Single cell GCaMP6s + clones were then picked into wells of a Matrigel-coated 96-well plate and expanded for further applications.

Cardiac differentiation of hPSCs via the GiWi method (Lian *et al.*, 2012)

Cardiac differentiation of hPSCs was performed when cells reach 80%-95% confluency. On day 0, cells were treated with 5-12 μM CHIR99021 (Selleckchem) in RPMI media for 18 hours, followed by a change with RPMI + 2% B27-insulin medium on day 1 (Note: Since B27-insulin supplement is not included in day 0 media as previously reported, increased cell toxicity is expected from day 0-1 and the remaining cell coverage should be 30-50% on day 1). Media was changed with RPMI + 2% B27-insulin again on day 2. On days 3 and 4, media is changed using 50% old media from the well with 50% fresh RPMI + 2% B27-insulin with the addition of 2 μM Wnt-C59 (Tocris) (Note: Migrating cells were observed on days 3 and 4. By day 4, cells should cover the whole well). Media was changed with RPMI + 2% B27 (Gibco) on day 5. Cells were then cultured in RPMI + 2% B27 with a media change every three days. Beating cells were observed at earliest on day 9. Once contractile function was observed, media was changed with RPMI + 2% FBS every three days.

Endoderm differentiation of hPSCs

Endoderm differentiation of hPSCs was performed when cells reach 80%-95% confluency. On day 0, cells were treated with 100 ng/mL Activin A and 2 μM CHIR99021 (Selleckchem) in RPMI media for 24 hours, followed by a change with RPMI + 2% FBS media containing 100 ng/mL Activin A and 5 ng/mL FGF2 on day 1 and day 2. On day 3, cells were fixed and stained against SOX17.

Ectoderm differentiation of hPSCs

To initiate ectoderm differentiation, hPSCs were passaged with LaSR basal medium (Lian *et al.*, 2014), which consists of Advanced DMEM/F12, 2.5 mM GlutaMAX, and 60 μg/ml ascorbic acid (Sigma, A8960), on day 0. Then cells were maintained in LaSR basal medium for 3-4 additional days before immunostaining against PAX6.

Flow cytometry

For the purpose of verifying the expression of GFP in knockin cell line, cells were dissociated into single cells with EDTA and followed by fixation with 1 % (v/v)

formaldehyde in PBS for 30 min at room temperature. The cells were then added into PBS containing 0.1 % (v/v) Triton X-100 and 0.5 % (v/v) BSA before flow cytometry analysis. Data were collected on a BD LSR Fortessa flow cytometer and analyzed using FlowJo.

Immunostaining

Cells were fixed with 4 % (v/v) formaldehyde in PBS for 15 min at room temperature and then immunostained with primary and secondary antibodies in PBS with 0.4 % Triton X-100 and 5 % non-fat dry milk. Nuclei were stained with Hoechst 33342. A Nikon Ti Eclipse epifluorescence microscope was used for imaging analysis.

Isoprenaline treatment

Brightfield and fluorescence videos of untreated cardiomyocytes were taken. Then, isoprenaline was added directly into the culture medium, and cells were incubated at 37°C for 5 min. Post treatment videos were then taken at set intervals following isoprenaline treatment. After video collection was complete, the media was changed with fresh culture media. .

Analysis of fluorescence intensity fluctuation

Each video corresponding to a specific isoprenaline concentration was split to a series of frames using ImageJ. For frequency analysis, five regions of interest (ROI) were selected in each video and the average intensity of each ROI was calculated in Time Series V3_0, generating a plot of time-tracing intensity. Frequency is determined as the reciprocal of average time between two adjacent peaks of intensity.

Analysis of contractile strain

The deformation and strain in CM clusters were calculated using a digital image correlation (DIC) technique in VIC-2D software (Correlated Solutions, Inc., SC, USA). The DIC technique calculates the motions of the cells by correlating the images of deformed cells with a reference image. To avoid accumulative error, an image in the diastolic period was used as reference. Each image was divided into blocks of pixels

(subsets) of 35×35 pixels (22.75 μm × 22.75 μm). The spacing of subsets was chosen to be 7 pixels (4.55μm). The cell cluster deformations were calculated by finding the maximum of the correlation value between image subsets in the deformed image and the reference image. Strain was calculated based on the derivative of the deformation. The maximum principal strain over time was extracted for 5 locations in each image series, respectively.

Statistical analysis

Numerical data are reported as mean ± standard deviation (SD) of the mean. The statistical significance between two sets of data was calculated using a two-tail Student's t-test. A value of $p<0.05$ was considered to be statistically significant.

References

Lian, X., Bao, X., Al-Ahmad, A., Liu, J., Wu, Y., Dong, W., Dunn, K. K., Shusta, E. V. and Palecek, S. P. (2014) 'Efficient Differentiation of Human Pluripotent Stem Cells to Endothelial Progenitors via Small-Molecule Activation of WNT Signaling', *Stem Cell Reports*, 3(5), pp. 804–816.

Lian, X. J., Hsiao, C., Wilson, G., Zhu, K. X., Hazeltine, L. B., Azarin, S. M., Raval, K. K., Zhang, J. H., Kamp, T. J. and Palecek, S. P. (2012) 'Robust cardiomyocyte differentiation from human pluripotent stem cells via temporal modulation of canonical Wnt signaling', *Proceedings of the National Academy of Sciences of the United States of America*, 109(27), pp. E1848–E1857.
